# Multifaceted Regulations of the Serotonin Transporter: Impact on Antidepressant Response

**DOI:** 10.3389/fnins.2019.00091

**Published:** 2019-02-12

**Authors:** Anne Baudry, Mathea Pietri, Jean-Marie Launay, Odile Kellermann, Benoit Schneider

**Affiliations:** ^1^INSERM UMR-S 1124, Paris, France; ^2^Université Paris Descartes, Sorbonne Paris Cité, UMR-S 1124, Paris, France; ^3^Hôpital Lariboisière, AP-HP, INSERM UMR-S 942, Paris, France; ^4^Pharma Research Department, Hoffmann-La Roche Ltd., Basel, Switzerland

**Keywords:** SERT, SSRIs, microRNAs, trafficking, phosphorylation, Na/K ATPase

## Abstract

Serotonin transporter, SERT (*SLC64A* for solute carrier family 6, member A4), is a twelve transmembrane domain (TMDs) protein that assumes the uptake of serotonin (5-HT) through dissipation of the Na^+^ gradient established by the electrogenic pump Na/K ATPase. Abnormalities in 5-HT level and signaling have been associated with various disorders of the central nervous system (CNS) such as depression, obsessive-compulsive disorder, anxiety disorders, and autism spectrum disorder. Since the 50s, SERT has raised a lot of interest as being the target of a class of antidepressants, the Serotonin Selective Reuptake Inhibitors (SSRIs), used in clinics to combat depressive states. Because of the refractoriness of two-third of patients to SSRI treatment, a better understanding of the mechanisms regulating SERT functions is of priority. Here, we review how genetic and epigenetic regulations, post-translational modifications of SERT, and specific interactions between SERT and a set of diverse partners influence SERT expression, trafficking to and away from the plasma membrane and activity, in connection with the neuronal adaptive cell response to SSRI antidepressants.

## Introduction

Serotonin (5-hydroxytryptamine, 5-HT) signaling in the central nervous system (CNS) modulates several physiological functions, including sleep, mood, anxiety, appetite, cognition as well as memory, and perception (for review see Olivier, 2015 and references therein), and in the periphery gut and platelet functions (Mercado and Kilic, 2010; Mawe and Hoffman, 2013). The precise extent and temporal dynamics of 5-HT signaling depend on control mechanisms that notably rely on the clearance of the released neurotransmitter by the high affinity serotonin transporter (SERT or 5-HTT), and to a lesser extent, the low affinity, high capacity organic cation transporters (OCTs) and the plasma membrane monoamine transporter (PMAT) at the cell surface, and the vesicular monoamine transporter (VMAT) in storage granules (Amphoux et al., 2006; Koepsell et al., 2007; Duan and Wang, 2010; Matthaeus et al., 2015).

**FIGURE 1:**
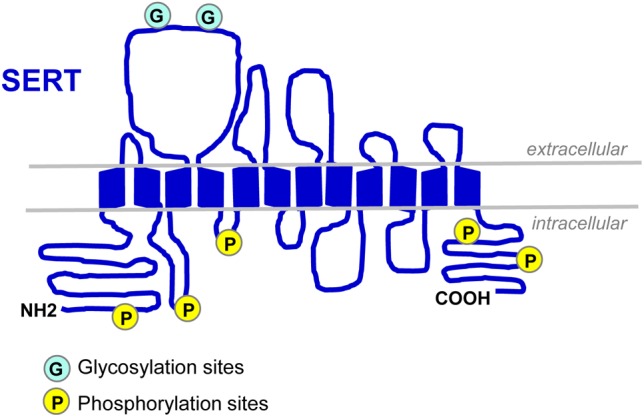
Schematic representation of SERT. SERT protein displays two N-linked glycosylation sites in the extracellular loop 2 (Launay et al., 1992; Tate and Blakely, 1994) and several sites of phosphorylation located in the N- and C-termini and in intracellular loops 1 and 2 (Vaughan, 2004; Sorensen et al., 2014).

Serotonin transporter (*SLC64A* for solute carrier family 6, member A4) belongs to the *SLC6* gene super family of Na^+^/Cl^-^-dependent transporters. The SERT encoding gene was first cloned from rat brain and basophilic leukemia cells in 1991 (Blakely et al., 1991; Hoffman et al., 1991). Two years after, the human SERT gene was cloned: it is present on chromosome 17q11.2 and contains 14/15 exons spanning around 40 kb (Ramamoorthy et al., 1993). In 1992, SERT protein was purified to homogeneity from human platelets (Launay et al., 1992). SERT is a 12 transmembrane domain (TMDs) protein containing two sites of N-linked glycosylation (Launay et al., 1992; Figure 1). This transporter is mainly located in cholesterol-rich membrane microdomains, also called lipid-rafts that act as platforms for the regulated assembly and functioning of signaling receptors and transporters (Allen et al., 2007). The N- and C-terminal regions of SERT dip into the cytosol and interact with several proteins that define, at least in part, the localization, stability and activity of SERT. Cytoplasmic domains located between TMDs also contain sites of post-translational modifications, showing that 5-HT transport is a highly regulated process.

Serotonin transporter assumes the active co-uptake of 5-HT and Cl^-^ ion using as the energy force the Na^+^ gradient created by the plasma membrane Na/K ATPase (Rudnick, 1977). It is generally admitted that SERT-mediated uptake of one 5-HT (a monovalent cation at physiological pH) with one Na^+^ and one Cl^-^ is electroneutral as the transport of the transmitter and ions is coupled to the efflux of one K^+^ ion (Rudnick and Nelson, 1978). However, SERT-mediated 5-HT uptake was shown to generate currents and to be electrogenic. This means the fixed stoichiometry of 5-HT and ions is probably not the only valid model for 5-HT transport and SERT may display ion channel-like property (for review, see De Felice, 2016 and references therein). In any case, in serotonergic neurons, serotonin uptaken by SERT adds to that synthesized *de novo* and increases the intracellular neurotransmitter pool. Three-Dimensional-Quantitative Structure-Activity Relationships studies allowed to show that SERT selectively uptakes specific 5-HT conformers with anti, -gauche and +gauche side-chain conformation, and to identify chemical determinants of the 5-HT molecule critical for 5-HT interaction with SERT (Pratuangdejkul et al., 2005, 2008).

Dysregulation of 5-HT signaling has been linked to several CNS-associated disorders such as depression, obsessive-compulsive disorder, anxiety disorders, and autism spectrum disorder (Murphy et al., 2004; Murphy and Lesch, 2008). Intensive researches have been carried out from the 50s to develop therapeutic compounds that antagonize SERT activity in order to maintain a tonic concentration of 5-HT at the synapse and/or in the surrounding milieu of serotonergic neurons. A particularity of the serotonergic system is to release 5-HT from extrasynaptic sites, the soma (Kaushalya et al., 2008; Trueta et al., 2012) and neuritic varicosities (Tork, 1990). 5-HT then acts as a volume transmitter involved in paracrine neuromodulation effects (Fuxe et al., 2007). In this context, drugs targeting SERT are serotonin reuptake inhibitors such as tricyclic inhibitors (e.g., imipramine), selective serotonin reuptake inhibitors (SSRIs, e.g., paroxetine and fluoxetine [Prozac^®^]), or compounds that lead to transport reversal (e.g., drugs of abuse such as amphetamines derivatives like MDMA, “ectasy”). However, the 3-week delay for beneficial effects of SSRIs treatment in patients with a depressive disorder [(Machado-Vieira et al., 2010) and references therein], and the fact that two thirds of the patients do not respond to antidepressants (Fava, 2003; Fekadu et al., 2009), reflect complex regulatory mechanisms of SERT that deserve considerations to better understand the functioning of SERT molecule and refine the options that would modulate 5-HT clearance by SERT in patients suffering from neuropsychiatric disorders. We will here review our current knowledge of the mechanisms that contribute to regulation of SERT activity and how specific regulation pathways render SERT responsive to antidepressant action.

## Genetic and Transcriptional Regulations of SERT Gene Expression

Several lines of evidence support that SERT expression and activity are influenced by genetic variations of SERT encoding gene. Variations in the serotonin-transporter-gene-linked polymorphic region (5-HTTLPR) located ∼1.2 kb upstream of the transcription start site have been shown to influence SERT expression and function. Lesch et al. (1996) found that the short promoter variant (S) containing 14 tandem repeats is associated with lower transcriptional activity, leading to reduced expression of SERT compared to the long (L) allelic variant composed of 16 repeats. Based on epidemiological studies, a link between such functional polymorphism in the SERT promoter and the susceptibility to life stress related depression has been proposed (Caspi et al., 2003). Ten years later, the identification of the single-nucleotide polymorphisms (SNPs) rs25531 (Hu et al., 2006) and rs25532 (Wendland et al., 2008) close to the 5-HTTLPR added allele variants that in combination with the L and S alleles contribute to the modulation of SERT expression and activity. Other mutations have also been described in SERT exon sequences (SERT I425V, G56A…) (Glatt et al., 2001; Prasad et al., 2005). Some of them lead to an increase of 5-HT uptake (Kilic et al., 2003) and/or alter SERT regulation by PKG and p38-MAPK signaling pathways (Prasad et al., 2005).

A second polymorphism region was evidenced in intron 2 (STin2) of the human SERT gene (Ogilvie et al., 1996). This region consists of a variable number of tandem repeats (VNTR) with 9 (STin2.9), 10 (STin2.10), or 12 (STin2.12) copies of a 16/17 bp element. Using mouse embryos or embryonic stem cells, the STin2 polymorphism was shown to function as transcriptional regulator (Fiskerstrand et al., 1999; MacKenzie and Quinn, 1999). Further *in vitro* analyses demonstrated that STin2 and 5-HTTLPR can contribute in concert to the gene expression (Ali et al., 2010) suggesting that combinations of VNTRs could modulate *in vivo* the level of expression of SERT, and thus the amount of SERT present at the plasma membrane and thereby modifying the efficacy of SERT-mediated 5-HT uptake.

In the 3′-untranslated (UTR) region of the SERT gene, two polyadenylation sites located 567 and 690 bp downstream of the stop codon have been reported, as well as a common SNP (rs3813034) present in the distal polyA signal (Battersby et al., 1999; Gyawali et al., 2010). This polymorphism of the 3′-UTR may influence the translation, localization, and stability of SERT mRNA. However, it is presently unknown how polymorphisms in the 3′-UTR of SERT mRNA (i.e., mutations, SNP, polyA tail length,...) impact on the capacity of (i) the translation machinery, (ii) RNA binding factors that pilot localization of mRNA in polarized cells, (iii) RNA binding proteins that stabilize the transcripts, and/or (iv) microRNAs that regulate mRNA expression, to interact with the SERT mRNA.

Since the complete characterization of SERT gene promoter in 1998 (Heils et al., 1998), a combination of positive and negative signals and factors were shown to influence SERT transcription in developing and adult brain serotonergic neurons of raphe nuclei. This notably includes the Pet-1 transcription factor that plays a critical role in the speciation, development and regulation of the serotonergic system (Hendricks et al., 1999; Goridis and Rohrer, 2002). A Pet-1 binding site is located upstream of the SERT encoding gene and Pet-1 binding was shown to increase SERT gene transcription *in vitro* (Hendricks et al., 1999). Lmx1b is another transcription factor that acts with Pet-1 to regulate the expression of SERT, as well as of other proteins related to the serotonergic system, including tryptophan hydroxylase (TPH) and VMAT. Selective inactivation of Lmx1b in serotonergic neurons of the raphe nuclei of adult mice leads to dramatic down-regulation of SERT, TPH, and VMAT expression, despite normal Pet1 level (Song et al., 2011).

Because of the presence of a cyclic AMP (cAMP) response element-like motif (CRE) in SERT promoter, cAMP signaling can also stimulate SERT gene transcription (Heils et al., 1998). In line with this, SERT transcription in the mouse midbrain is influenced by the dark/light alternation, with higher levels of SERT mRNA during the dark phase than during the light phase. In mouse, the expression of SERT mRNA follows a 24 h oscillation rhythm and depends on the clockwork system as well as the ATF4 transcription factor that sustains circadian oscillations of CRE-mediated gene expression and binds the CRE site of the SERT promoter (Ushijima et al., 2012). At the mRNA and protein level, the ATF4 transcription factor itself was shown to vary in the mid-brain of mice according to a 24-h rhythm with higher levels in the dark phase (Ushijima et al., 2012). Such time-dependent variations of ATF4 amount depend on the clockwork system as they are no longer observed in *Clock* mutant mice (Ushijima et al., 2012). With the help of the 1C11 inducible neuronal stem cell line that differentiates into serotonergic neuronal cells (1C11^5-HT^) (Buc et al., 1990; Mouillet-Richard et al., 2000), our laboratory provided evidence that the onset of SERT protein and activity obeys to a mechanism linked to the neuronal differentiation of 1C11 stem cells (Launay et al., 2006). SERT encoding mRNAs are present at the stem cell stage, but are dormant. Exposure of 1C11 precursors to dibutyril cyclic AMP (dbcAMP) to recruit the serotonergic neuronal program does not affect SERT mRNA level, but promotes a rise in the length of SERT mRNA polyA tail (+200 base pairs) that precedes SERT translation and trafficking to the plasma membrane (Launay et al., 2006). Yammamoto et al. (2013) also reported dbcAMP-dependent induction of SERT expression along neural differentiation of SERT-transfected RN46A cells derived from embryonic rat raphe nuclei that depended on protein kinase A (PKA) activity and was further associated with reduced degradation rate of the SERT protein. cAMP signaling thus contributes to the control of SERT expression at multiple levels: gene transcription by acting on the SERT promoter through CRE regions, polyadenylation of the SERT mRNA along neuronal differentiation that unlocks the translation of SERT, and increased stability of the SERT protein.

Glucocorticoid signaling was also shown to positively influence SERT gene transcription in human lymphoblastoid cells (Glatz et al., 2003) as well as in serotonergic neurons (Lau et al., 2013). In the latter case, glucocorticoid-induced SERT expression likely underlies the contribution of the hypothalamo-pituitary-adrenal axis (also called stress axis) to modulation of serotonergic functions in raphe nuclei (Lau et al., 2013) and the possible protective function of glucocorticoids in the regulation of emotional behavior as shown by reduced exploratory behavior in zebrafish mutants defective for the expression of glucocorticoid receptor (Ziv et al., 2013). Of note, dysregulation of the stress axis indeed represents a hallmark of major depression in human patients (Pittenger and Duman, 2008; Keller et al., 2017).

A growing body of evidence indicates a role of the immune system and inflammation in the pathophysiology of neurological disorders, including depression (Furtado and Katzman, 2015; Robson et al., 2017; Leonard, 2018), anxiety (Salim et al., 2012; Reader et al., 2015) or autism spectrum disorders (Gesundheit et al., 2013). Among cytokines and interleukins produced along the inflammation process, interleukin 1 (IL1) upregulates SERT translation in JAR human placental choriocarcinoma cells (Ramamoorthy et al., 1995), possibly through signaling pathways involving MAP kinases and NF-kappaB transcription factor (Kekuda et al., 2000). On the contrary, interleukin 6 (IL6) was shown to negatively act on SERT gene transcription. SERT expression in the hippocampus is reduced upon mouse treatment with IL6, which can be counteracted upon inhibition of the STAT3 transcription factor (Kong et al., 2015). One would expect an anti-depressive action of IL6 treatment that contrasts with sustained elevation of IL6 levels in patients with major depressive disorders and depressive-like phenotype of mice injected with recombinant IL-6 (Sukoff Rizzo et al., 2012). Further investigations are needed to delineate how IL6-mediated SERT reduction leads to depressive states.

## Regulation of SERT Trafficking

A second layer of SERT regulations concerns the trafficking and bioavailability of the serotonin transporter at specific plasma membrane subdomains to achieve localized clearance of 5-HT.

First evidence comes from the observation that depletion of cholesterol in membranes of human embryonic kidney 293 cells stably expressing rat SERT decreases SERT activity (Scanlon et al., 2001). Magnani et al. (2004) then reported a partitioning of SERT molecules to a subpopulation of lipid rafts of the plasma membrane in the rat brain. Cholesterol present in the membrane bilayer affects the conformation of SERT and its transport kinetics parameters through binding to the SERT conserved cholesterol site 1 located in a hydrophobic groove between TMD1a, TMD5, and TMD7 (Laursen et al., 2018). Beyond cholesterol, SERT activity, and SERT trafficking to the plasma membrane could be influenced by phosphatidylinositol-4,5-biphosphate (PIP2), whose binding to SERT in the endoplasmic reticulum (ER) was reported to drive the oligomerization of SERT, to target SERT homo-oligomers to the cell surface and to positively impact on SERT activity (Anderluh et al., 2017). How PIP2 level impact on SERT present at the plasma membrane is presently unknown, since PIP2 levels drop down at the plasma membrane due to its conversion into inositol triphosphate (IP3) and diacylglycerol (DAG) by phospholipase C.

From a structural point of view, the carboxyl terminus of SERT is a critical domain of the transporter necessary for its delivery to the plasma membrane. SERT molecules truncated for 17–30 amino-acid residues in the C-terminal region in fact lack mature glycosylation and fail to reach the cell surface during the synthesis/secretory process (Larsen et al., 2006; Nobukuni et al., 2009). Specific subdomains of the C-terminus would influence human SERT folding and the formation of a docking site for a coat protein (COPII) component necessary to export SERT from the ER to the plasma membrane (El-Kasaby et al., 2010), and/or limit the influence of Heat Shock Proteins on SERT retention in the ER (El-Kasaby et al., 2014). The study by Ahmed et al. (2008) reinforces the role of the C-terminus of SERT for translocation of the transporter from intracellular compartments to the plasma membrane. Starting from the observation that the density of SERT molecules is reduced at the cell surface of platelets when plasmatic concentrations of 5-HT are elevated, the authors provided evidence that this high level of 5-HT causes 5-HT transamidation (serotonylation) of the small GTPase Rab4 within platelets, leading to the stabilization of Rab4 in its active GTP-bound form, binding of Rab4 to the cytoplasmic C-terminus part of SERT and retention of the transporter in intracellular compartments (Ahmed et al., 2008). Such impact of an excess of 5-HT on SERT trafficking in platelets can be viewed as a way to maintain an external tonic 5-HT concentration for regulating/amplifying blood functions, such as platelet aggregation (Walther et al., 2003). On the other hand, the direct interaction of the C-terminus of SERT with active integrin αIIbβ3 enhances SERT activity that correlates with increased SERT expression at the surface of transfected HEK293 cells (Carneiro et al., 2008). Refining the functional interaction between integrin β3 and SERT, Mazalouskas et al. (2015) provided evidence for tight modulation of the activity of a subpopulation of SERT molecules by the αvβ3 integrin receptor subtype in the midbrain at serotonergic synapses. Partial neuronal depletion of integrin β3 subunit in mice reduces SERT-mediated 5-HT uptake in midbrain synaptosomes by scaling down the population size of active SERT molecules (Mazalouskas et al., 2015). In this context, polymorphism in human integrin β3 has been suggested to impact on the responsiveness of some patients to SSRIs. Interestingly, decrease of SERT cell surface localization and 5-HT uptake was reported upon interaction of Nitric Oxide synthase, through its PDZ domain, with SERT C-terminus (Chanrion et al., 2007). Thus, SERT trafficking to or away from the plasma membrane depends on the protagonists interacting with the carboxy-terminal domain of SERT.

Association of diverse proteins to SERT regions distinct from the C-terminus has also been shown to influence the trafficking of SERT. This includes the secretory carrier membrane protein 2 (SCAMP2) that interacts with the N-terminus of SERT, leading to subcellular redistribution of SERT, with a reduction of its density at the cell surface (Muller et al., 2006). Some other proteins, whose SERT binding site has not yet been identified, also impact on SERT localization. α-synuclein, a protein mainly known for its implication in Parkinson’s disease, binds SERT through direct protein-protein interactions via the non-Aβ-amyloid component domain of the α-synuclein protein and promotes SERT internalization, accounting for reduced 5-HT uptake (Wersinger et al., 2006; Wersinger and Sidhu, 2009). The membrane glycoprotein M6B, a proteolipid notably expressed in neurons and oligodendrocytes in the brain, interacts with SERT, down-regulates its trafficking to and/or stability at the plasma membrane, and thereby decreases SERT-mediated serotonin uptake in transfected cells (Fjorback et al., 2009). Formation of a hetero-complex between SERT and ASCT2 (for alanine-serine-cysteine-threonine2), a solute carrier 1 family member co-expressed with SERT in serotonergic neurons and involved in the plasma membrane transport of neutral amino acids, also reduces cell surface localization of SERT and SERT activity (Seyer et al., 2016). Although the mechanism remains elusive, interaction of SERT with the vesicle-associated membrane protein 2 (VAMP-2), a SNARE protein involved in the vesicle fusion with the plasma membrane, positively influences SERT translocation to the plasma membrane and thereby SERT function (Muller et al., 2014).

A complex signaling network involving kinases and phosphatases has also been shown to largely influence the presence of SERT at the cell surface. Exploiting the non-neuronal HEK-293 cell system, Ramamoorthy et al. (1998) reported the internalization of the transfected human SERT through a protein kinase C (PKC)-dependent pathway, leading to reduced 5-HT uptake. PKC-dependent internalization of SERT would depend on SERT interaction with the LIM domain adaptor protein Hic-5 (Carneiro and Blakely, 2006). The same group further showed in rat basophilic leukemia 2H3 cells that protein kinase G (PKG)-connected pathways enhance SERT surface trafficking (Zhu et al., 2004). Another protagonist that positively influences SERT presence at the plasma membrane is the Akt/PKB protein kinase, as the inhibition of Akt1 and Akt2 reduces SERT export to the plasma membrane, possibly through post-translational modification of SERT by phosphorylation (Rajamanickam et al., 2015). The Ca^2+^-activated protein phosphatase calcineurin was shown to influence both *in vitro* and *in vivo* SERT trafficking to the plasma membrane (Seimandi et al., 2013): both catalytic and regulatory subunits of calcineurin bind SERT C-terminus and the physical association of calcineurin with SERT depends on calcineurin phosphatase activity. Calcineurin interaction with SERT was shown to prevent reduction of SERT uptake activity induced by PKC-mediated SERT phosphorylation. Unexpectedly, constitutive calcineurin activation in mice generates antidepressant-like effects, that is, reduced immobility in the forced swim test (Seimandi et al., 2013). How calcineurin contributes to antidepressant effects remains largely elusive and needs extensive investigations (Seimandi et al., 2013).

Supporting the idea that modification of SERT trafficking could underlie the beneficial action of antidepressants, Schloss’s laboratory showed that exposure of 1C11-derived serotonergic neurons to citalopram, and to a lesser extent, fluoxetine, paroxetine, and sertraline, reduces the level of SERT molecules present at the neuronal cell surface (notably in neurites) through SERT internalization and redistribution to the cell body (Lau et al., 2008; Kittler et al., 2010; Matthaus et al., 2016). The consequence is a reduction of 5-HT uptake and increase of the external 5-HT concentration. How SSRI antidepressants promote endocytosis of SERT molecules and their redistribution to the soma is unknown and needs further investigations.

## Regulation of SERT Uptake Activity

Beyond the impact of phosphorylations on SERT trafficking/residence at the plasma membrane, phosphorylation steps also impact on SERT uptake activity. In SERT-transfected CHO cells, activation of p38-MAP kinase downstream from PKG in adenosine receptor-coupled signaling pathways with a critical implication of the protein phosphatase 2A was shown to up-regulate SERT catalytic activity (Zhu et al., 2005). Although it remains controversial (Andreetta et al., 2013; Schwamborn et al., 2016), implication of p38-MAPK in the upregulation of SERT activity induced by IL1β and TNFα was observed in the mouse midbrain and striatal synaptosomes (Zhu et al., 2006, 2010). Ramamoorthy et al. (2007) further provided evidence that PKG activation does not affect SERT surface abundance, excluding that the increase of SERT activity originates from enhanced trafficking of SERT to the plasma membrane. In that study, the authors firstly identified Thr276 of the SERT molecule, as a site whose phosphorylation by PKG augments the Vmax of SERT-mediated 5-HT uptake (Zhang et al., 2016). In addition to Thr276, five other residues located in the N- and C-termini and in intracellular loop 1 and 2 of the SERT molecule were identified by liquid-chromatography-tandem mass spectrometry as phosphorylation sites. These are Ser149/Ser277/Thr603 for PKC, Ser13 for CaMKII, and Thr616 for p38MAPK (Sorensen et al., 2014). Tyrosine phosphorylations of SERT by signaling pathways coupled to Src kinase activity, including Syk, were also shown to positively influence SERT-mediated 5-HT accumulation in dense granules of platelets (Zarpellon et al., 2008; Pavanetto et al., 2011), but Tyr residue(s) involved remain(s) to be identified.

In human placenta choriocarcinoma cell line (Sakai et al., 1997) as well as in rat platelets (Jayanthi et al., 2005), activation of PKC by phorbol esters triggers rapid inhibition of SERT uptake activity associated with decreased Vmax and sometimes increased Km. Such reduction of SERT intrinsic activity depends on PKC-mediated phosphorylations of SERT present at the plasma membrane on Ser residues. This step precedes the PKC-mediated internalization of SERT, due to additional phosphorylations on Thr residues, thus sustaining strong reduction of SERT activity because of enhanced endocytosis of SERT (Jayanthi et al., 2005; Carneiro and Blakely, 2006). The direct phosphorylation of SERT by PKC has been questioned in a study by Sakai et al. (2000), which showed that the negative action of PKC on SERT activity rather depends, at least in part, on the disruption of F-actin cytoskeleton and morphological cell changes.

**FIGURE 2:**
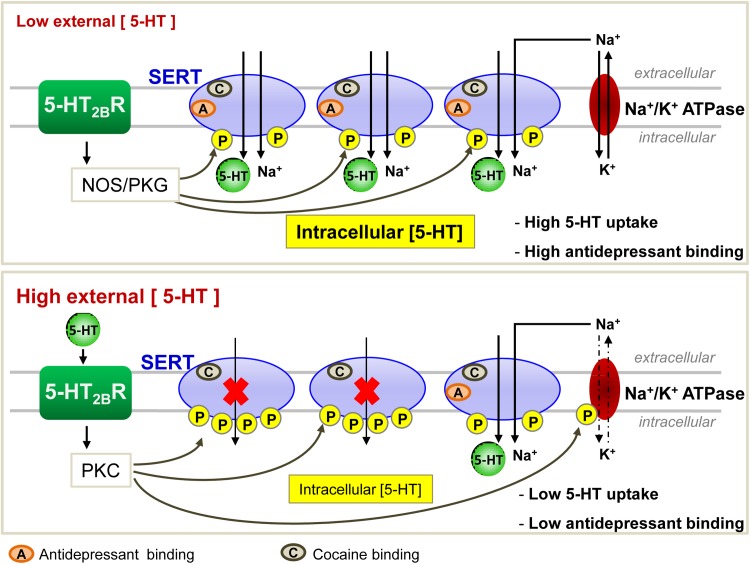
Regulation of SERT function by the serotonergic receptor 5-HT_2B_ in 1C11^5-HT^ neuronal cells. At low 5-HT concentration, 5-HT_2B_Rs through its NO signaling coupling ensure a basal phosphorylation level of SERT, endowing it with an optimal 5-HT uptake activity. All SERT molecules are able to bind antidepressants. At high level of 5-HT, 5-HT_2B_R-dependent protein kinase C (PKC) couplings provoke hyperphosphorylation of SERT that impairs its ability to uptake 5-HT. Under these conditions, only 1/3 of SERT molecules bind antidepressants.

Interestingly, in HEK-293 cells transfected with SERT, 5-HT counteracts PKC-dependent SERT phosphorylation and rescues SERT uptake activity (Ramamoorthy and Blakely, 1999). With the help of prefrontocortical synaptosomes, Awtry and colleagues further report that PKA, downstream nicotinic acetylcholine receptors-coupled signaling pathways, increases SERT activity. Nicotine-induced increase of 5-HT levels in the brain prefrontal cortex may account for the effects of nicotine on behaviors such as cognition, reward and memory (Awtry et al., 2006). Through pharmacological approaches using agonists and antagonists of serotonergic 5-HT_2B_ receptor (5-HT_2B_R) subtype, our laboratory showed that 5-HT_2B_R signaling underlies the action of 5-HT on the control of SERT uptake activity and its energy source, the Na/K ATPase electrogenic pump (Figure 2; Launay et al., 2006). In serotonergic 1C11^5-HT^ neuronal cells as well as in primary neuronal culture derived from embryonic raphe nuclei, 5-HT_2B_R governs the phosphorylation state of both SERT and Na/K ATPase. At low 5-HT concentration [1–2 nM, as in *in vivo* physiological conditions (Brand and Anderson, 2011)], the intrinsic 5-HT_2B_R coupling to NOS and the subsequent activation of PKG ensure SERT phosphorylation to basal level that correlates with maximal 5-HT uptake. In these conditions, SERT molecules are fully competent for binding cocaine and paroxetine. In excess of 5-HT, 5-HT_2B_R coupling to the IP_3_/PKC pathway promotes additional phosphorylation of SERT and in parallel enhances the phosphorylation level of the Na/K ATPase. PKC-mediated phosphorylation of Ser residues in the N-terminus of the alpha subunit of Na/K ATPase was shown to reduce pump activity through mechanisms that remain elusive, but that could relate to the acquisition of an altered structure caused by the phosphorylations and/or the endocytosis of the Na/K ATPase (for review, see Poulsen et al., 2010 and references therein). In any case, SERT hyperphosphorylation combined to the phosphorylation rise of Na/K ATPase reduces 5-HT transport efficacy (Figure 2). Importantly, the population of SERT molecules hyperphosphorylated upon 5-HT_2B_R stimulation loose their ability to bind paroxetine. As hyperphosphorylated SERT keeps its capacity to bind cocaine at the plasma membrane of neuronal cells, reduced SERT uptake activity and reduced SERT binding of SSRIs are not attributable to SERT internalization induced by excess of 5-HT. The impact of 5-HT and 5-HT_2B_R signaling on SERT uptake activity can be viewed as a feedback loop that *in fine* permits keeping extracellular serotonin at tonic concentrations necessary for regulating sleep, mood, appetite, and some cognitive functions. In addition to influencing SERT function, 5-HT_2B_R-dependent control of Na/K ATPase activity would have a broader action by impacting other transporters whose activity depends on this electrogenic pump. Of note, the fact that stimulation of 5-HT_2B_R promotes SERT hyperphosphorylation and thereby reduces SERT sensitivity to antidepressants may explain, at least partly, the resistance of some patients to antidepressant treatment and/or the time-delay of the antidepressant response. Whatever their phosphorylation state, the half-life of SERT molecules present at the plasma membrane of serotonergic neurons is unknown. Considering that hyperphosphorylated SERT, which is non-competent for antidepressant recognition and thereby confers resistance to antidepressant action, may stay at the surface of serotonergic neurons even if external 5-HT concentration collapses (depressive situations), binding of SSRIs to SERT will depend on the neo-synthesis and trafficking of non-, or poorly phosphorylated SERT to the plasma membrane, a process whose kinetics could take several days. Further investigations are therefore needed to appreciate the stability of the different phosphorylated forms of SERT at the plasma membrane and their turnovers in fully integrated serotonergic systems.

## Epigenetic Regulation of SERT Expression and Activity

Over the past decade, evidence has accumulated that SERT expression and thereby its functions are also governed by epigenetic regulation, including DNA methylation, histone modifications and microRNAs. In this respect, it has been shown that in lymphoblast cell lines, methylation of CpG island nearby the untranslated exon1A of SERT encoding gene is associated with reduced levels of SERT mRNA, an effect that depends on 5HTTLPR genotype (Philibert et al., 2007). Accordingly, Kinnally et al. (2010) reported that in rhesus macaques, the short allele rh5HTTLRP presented higher level of CpG methylation than the longer one, which was associated with reduced SERT expression in peripheral blood mononuclear cells. Further, using a luciferase reporter construct, Wang et al. showed *in vitro* reduced SERT transcription when its promoter is methylated in JAR cells (Wang et al., 2012). However, a role of DNA methylation in SERT regulation is still at the heart of debates since Wankerl et al. (2014) observed in their cohort of 133 healthy young participants that variations of SERT mRNA level appear to be independent of DNA methylation profiles within the SERT CpG island.

In few reports, modifications in the acetylation status of histones were found to impact on SERT expression. Histone deacetylases (HDAC) inhibitors [butyrate, trichostatin A (TSA)] or knockdown of HDAC2 (but not HDAC1) by RNA interference in the human intestinal epithelial cell line Caco-2 reduced SERT mRNA and protein levels (Gill et al., 2013). By contrast, in several tumor cell lines (HD11, HepG2, THP-1...), addition of TSA or siRNA-mediating silencing of HDAC1 enhanced the expression of SERT mRNA, leading to an increase of 5-HT uptake (Phi van et al., 2015).

**FIGURE 3:**
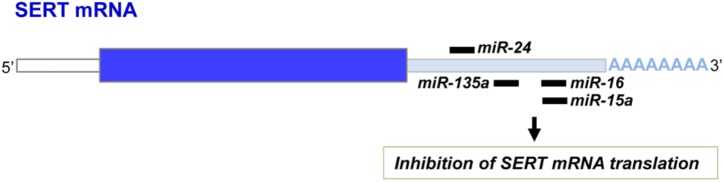
miR-16, miR-15a, miR-135a, and miR-24 binding sites on the 3′ untranslated region of the SERT mRNA.

**FIGURE 4:**
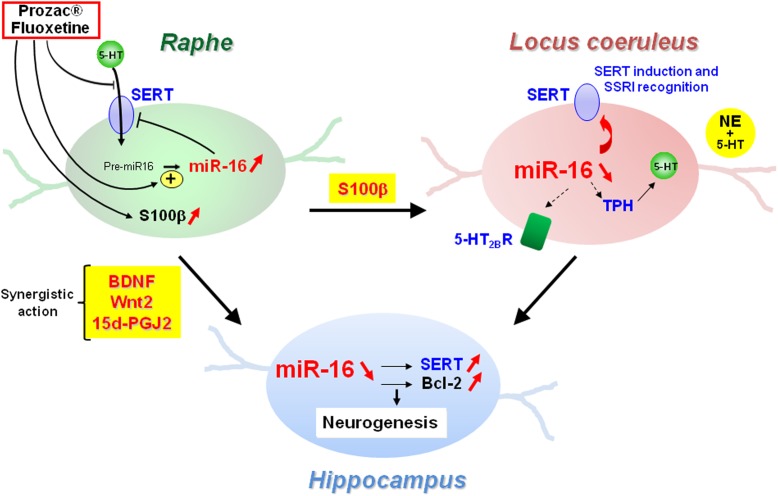
Central role of miR-16 in the adaptive response of neurons from raphe, locus coeruleus, and hippocampus to SSRI antidepressant treatment.

Another layer of epigenetic control of SERT level relies on microRNAs that are small non-coding RNA able to interact with the 3′ untranslated region (3′UTR) of the mRNA of target genes and block their translation. In 2010, our laboratory identified the microRNA miR-16 as a regulator of SERT expression (Baudry et al., 2010; Figure 3). The level of miR-16 is low in serotonergic neurons (1C11^5-HT^ cells or raphe nuclei), where SERT is expressed. In contrast, miR-16 is abundant in noradrenergic neurons (1C11^NE^ cells or locus coeruleus) and prevents the translation of SERT mRNA. We established that variation of miR-16 level sustains the antidepressant action of fluoxetine (Baudry et al., 2010; Launay et al., 2011). In mice, fluoxetine increases miR-16 in serotonergic neurons of raphe nuclei, which in turn decreases SERT protein level as observed in Prozac^®^-treated patients (Benmansour et al., 2002), thus increasing external 5-HT concentration. From a mechanistic point of view, the rise of miR-16 induced by fluoxetine in serotonergic neurons results from an increased maturation of pri/pre-miR-16 through a GSK3β-dependent process. Moreover, the fluoxetine action on raphe creates a dialog between raphe and locus coeruleus, and between raphe and hippocampus:

(i) Fluoxetine provokes the release of the neurotrophic factor S100β by serotonergic neurons that acts on noradrenergic neurons of the locus coeruleus. S100β signaling reduces miR-16 level in noradrenergic neurons, which unlocks the expression of SERT, but also of other serotonergic functions, including the expression of TPH. Noradrenergic neurons acquired a mixed noradrenergic/serotonergic phenotype and thus become a new source of serotonin in the brain (Baudry et al., 2010; Figure 4).
(ii) Fluoxetine also provokes the secretion of BNDF, Wnt2, and 15dPGJ2 by serotonergic neurons that act in synergy on another raphe-connected structure, the hippocampus. The cocktail of the three molecules decreases miR-16 levels in the hippocampus, which again unlocks SERT expression, but also enhances the level of a second miR-16 target, the anti-apoptotic factor bcl2, and sustains neurogenesis (Launay et al., 2011). Of note, the levels of BDNF, Wnt2, and 15dPGJ2 are augmented *in vivo* not only in the CSFs of mice but also in patients treated with Prozac^®^ (Launay et al., 2011).

Finally, using mouse models of depression, we observed that the upregulation of miR-16 in raphe or the downregulation of miR-16 in the locus coeruleus (Baudry et al., 2010) or in the hippocampus (Launay et al., 2011) provoke behavioral responses that compare to those induced by fluoxetine. Accordingly, Yang et al. (2017) reported that miR-16 administration in raphe nuclei or intragastric injections of fluoxetine in chronic unpredicted mild stress model rats for 3 weeks lead to similar improvement of depressive behavioral changes.

Can the fluoxetine-induced dialogs between raphe and locus coeruleus, or raphe and hippocampus account for the time-delay of action of antidepressants? Depression seems strongly related to a loss in the number of neurons and synapses in several brain structures, including the hippocampus and prefrontal cortex (Drevets et al., 1997; Hercher et al., 2009). Post-mortem studies indeed revealed hippocampal atrophy in cases of major depressive disorder, which can be reversed by antidepressant treatment (Duman, 2004; MacQueen and Frodl, 2011) through stimulation of hippocampal neurogenesis and synaptogenesis (Duman, 2004; Boldrini et al., 2009). Of note, these processes are slow. In response to antidepressants, the neuronal plasticity of noradrenergic neurons, but more likely the hippocampal neurogenesis orchestrated by the raphe nuclei, might account for the delay in the action of antidepressants.

Nevertheless, the picture is likely more complicated. SERT mRNA occurs in two alternative polyadenylated forms: a short form and a ∼125 bp longer form, which is associated with reduced anxiety-related behavior (Gyawali et al., 2010; Hartley et al., 2012). miR-16 blocks translation of both polyadenylated forms of the SERT mRNA (Yoon et al., 2013). However, Yoon et al. showed that the miR-16 regulation of SERT expression was modulated by a RNA binding protein hnRNPK after S100β addition in rat C6 astroglioma cells and RN46A brain raphe cells. S100β-induced Src kinases phosphorylate hnRNPK, which in turn displaces miR-16 from long polyadenylated SERT mRNA and allows its translation (Yoon et al., 2013). Thus, hnRNPK adds another layer for regulation of SERT expression specific to long polyadenylation forms of SERT mRNAs.

In addition to miR-16, other microRNAs were found to interact with the 3′UTR of the SERT mRNA and inhibit its translation (Figure 3). Among those, miR-15a, which belongs to the same cluster as miR-16, was shown to regulate SERT expression in the rat RN46A serotonergic cell line as well as in human JAR cells (Moya et al., 2013). In 2014, Issler and colleagues evidenced that viral-mediated overexpression of another miR, miR-135a, in mouse raphe induces a decrease of SERT protein level and reduces adverse effects of chronic social defeat. Conversely, miR-135a knockdown leads to a raise of SERT expression, an increase of anxiety-like behavior and a reduced response to SSRIs. Upon SSRI antidepressant administration to mice, upregulation of miR-135a level was observed in raphe nuclei (Issler et al., 2014). As for miR-16, increase of miR-135a induced by antidepressants in serotonergic neurons will repress SERT expression and thereby SERT-mediated serotonin uptake, thus contributing to the beneficial effect of antidepressants. miR-24 was identified as a regulator of SERT expression in intestinal mucosa epithelial cells (Liao et al., 2016). Patients suffering of irritable bowel syndrome (IBS) as well as a IBS mouse model display high levels of miR-24 and correlatively decreased SERT expression. Treatment of IBS mice with a miR-24 inhibitor increases SERT protein level and alleviates intestinal pain and inflammation (Liao et al., 2016). More recently, miR-195 and miR-322, in addition to miR-15 and miR-16, were found to inhibit SERT expression after transfection in smooth muscle cells (Gu et al., 2017).

Given the above findings, which receptors and downstream signaling pathways regulate the level of SERT-targeting microRNAs upon antidepressant action remain to be identified. Finally, it will be also critical to evaluate how antidepressants, by modulating microRNA expressions, modify the neuronal phenotype and, as for miR-16, promote changes of neurotransmission in raphe-connected brain regions.

## Conclusion

Since the enunciation in the 50s of the “monoamine theory of affective disorders” that stipulates decreased levels of bioamines in depressed patients, and the consecutive pharmaceutical development of SSRI antidepressants in the 60s, how SSRIs exert their antidepressant action remained largely obscure for almost 50 years. By showing that fluoxetine (Prozac^®^) binding to SERT not only blocks 5-HT uptake in serotonergic neurons, but also creates a new source of serotonin in raphe nuclei-connected locus coeruleus through modulations of miR-16 level, a huge breakthrough has been made in our understanding of the mode of action of some SSRI antidepressants that notably relies on the neuronal plasticity of noradrenergic and hippocampal neurons (Baudry et al., 2010; Launay et al., 2011). However, it is clear that a consequent fraction of depressed patients is hyporesponsive to SSRI antidepressants. The absence of antidepressant response likely relates to a molecular state of SERT incompetent for SSRI recognition. We illustrate in this review that multiple factors, including SERT polymorphism, post-translational modifications of SERT by phosphorylations, SERT partners, or SERT trafficking, influence the presence of SERT at the plasma membrane and competency to bind SSRI antidepressants. In this context, to predict the success of a SSRI treatment in depressed patients, the clinical rational would be first to determine SERT status in those patients. Even if non-invasive imaging techniques permit to delineate the density of SERT molecules in the human brain (Meyer, 2007), post-translational modifications of SERT cannot be addressed. Interestingly, SERT molecules expressed by blood platelets display pharmacological properties highly comparable to those of SERT in the CNS, in terms of 5-HT uptake and sensitivity to antidepressants (Tuomisto and Tukiainen, 1976; Tuomisto et al., 1979; Da Prada et al., 1988; Launay et al., 2006). As the SERT state in blood platelets would likely mirror the SERT state in serotonergic neurons of raphe nuclei, characterizing SERT post-translational modifications in blood platelets would be helpful to predict the response or refractoriness of depressed patients to one type of SSRIs prior to proposing a personalized medicine tailored to each individual depressed patient.

## Author Contributions

All authors listed have made a substantial, direct and intellectual contribution to the work, and approved it for publication.

## Conflict of Interest Statement

J-ML has non-financial competing interests with Hoffmann-La Roche Ltd., laboratories. He acts as an expert witness for Hoffmann-La Roche Ltd., laboratories. This does not alter his adherence to all Frontiers in Neuroscience policies. The remaining authors declare that the research was conducted in the absence of any commercial or financial relationships that could be construed as a potential conflict of interest.
